# Preservation of zebrafish genetic resources through testis cryopreservation and spermatogonia transplantation

**DOI:** 10.1038/s41598-019-50169-1

**Published:** 2019-09-25

**Authors:** Zoran Marinović, Qian Li, Jelena Lujić, Yoshiko Iwasaki, Zsolt Csenki, Béla Urbányi, Goro Yoshizaki, Ákos Horváth

**Affiliations:** 1 Department of Aquaculture, Szent István University, Páter Károly u. 1., H-2100 Gödöllő, Hungary; 2 Department of Marine Biosciences, Tokyo University of Marine Science and Technology, 108-8477 Tokyo, Japan

**Keywords:** Animal biotechnology, Stem-cell biotechnology, Ichthyology

## Abstract

Zebrafish is one of the most commonly used model organisms in biomedical, developmental and genetic research. The production of several thousands of transgenic lines is leading to difficulties in maintaining valuable genetic resources as cryopreservation protocols for eggs and embryos are not yet developed. In this study, we utilized testis cryopreservation (through both slow-rate freezing and vitrification) and spermatogonia transplantation as effective methods for long-term storage and line reconstitution in zebrafish. During freezing, utilization of 1.3 M of dimethyl sulfoxide (Me_2_SO) displayed the highest spermatogonia viability (~60%), while sugar and protein supplementation had no effects. Needle-immersed vitrification also yielded high spermatogonia viability rates (~50%). Both optimal slow-rate freezing and vitrification protocols proved to be reproducible in six tested zebrafish lines after displaying viability rates of >50% in all lines. Both fresh and cryopreserved spermatogonia retained their ability to colonize the recipient gonads after intraperitoneal transplantation of *vasa::egfp* and *actb:yfp* spermatogonia into wild-type AB recipient larvae. Colonization rate was significantly higher in *dnd*-morpholino sterilized recipients than in non-sterilized recipients. Lastly, wild-type recipients produced donor-derived sperm and donor-derived offspring through natural spawning. The method demonstrated in this study can be used for long-term storage of valuable zebrafish genetic resources and for reconstitution of whole zebrafish lines which will greatly improve the current preservation practices.

## Introduction

Zebrafish *Danio rerio* is a well-established, and one of the most widely used vertebrate model organisms in biomedical, developmental and genetic research^1^ mostly due to its small size, ease of culture, high fecundity, transparent embryos, 70% homology between the zebrafish and human genomes, as well as good annotation of the genome^2,3^. With extensive studies utilizing this model fish, several thousands of transgenic lines of zebrafish have been created by molecular biology tools (such as ZFNs, TALENs, CRISPR/Cas9) during the recent decades^2,3^. Furthermore, as the molecular methods are improved and refined along with the increasing needs in experimental designs, the number of transgenic lines is only going to increase. This trend is leading to great concerns in the storage and maintenance of these transgenic lines. Current practices are mostly limited only to keeping breeding colonies which requires a significant amount of space, is labor- and cost-demanding, but is also vulnerable to pathogen infections^3,4^. Therefore, there is a great need for improving storage conditions for such valuable genetic resources.

Cryopreservation is a method that allows the storage of genetic resources for an indefinite period of time in liquid nitrogen. Sperm cryopreservation protocols were developed for several hundreds of fish species so far (reviewed by Asturiano *et al*.^5^). However, there is a great shortcoming of this method since so far only spermatozoa can be successfully cryopreserved in fish, and effective methods for freezing of eggs and embryos have not yet been developed due to their high yolk content and structural complexity. Even though some progress has been made in cryopreservation of zebrafish embryos^6^, these methods still do not lead to the production of functional embryos which will be able to reconstitute a whole zebrafish line. Additionally, many protocols for freezing of zebrafish sperm yielded low and very variable post-thaw motility and fertilization rates^7–9^, thus limiting the applicability of sperm cryopreservation in reconstitution of zebrafish lines. Recently, the study of Matthews *et al*.^10^ standardized the sperm cryopreservation process to a great extent and demonstrated that reliable and reproducible results can be obtained for a large number of transgenic zebrafish lines. However, the absence of methods for conservation of female genetic resources still remains.

Due to the reasons above, we have focused on spermatogonial stem cells (SSCs) in this study. These are the baseline cells of spermatogenesis with a specific function to both self-renew and differentiate into later-stage germ cells and finally into spermatozoa^11^. Their ability to colonize recipient gonads after intraperitoneal transplantation, proliferate and differentiate into functional gametes (either spermatozoa or eggs depending on the sex of the recipient) after cryopreservation and to produce donor-derived offspring by recipients has been demonstrated by several studies^12–14^. Additionally, the study of Seki *et al*.^4^ demonstrated that this approach can be very effective in the conservation of genetic resources and reconstitution of important lines in valuable laboratory animals (medaka *Oryzias latipes*).

The surrogate production technology poses several challenges during its implementation. Even though transplanted SSCs have the ability to colonize the recipient gonads, a large number of the autologous germ cells still remain. In fact, the majority of gametes derived from such recipients are autologous^14^. Due to this reason, sterile recipients became imperative, and several strategies were developed. Hybrids between different species or triploid individuals are generally sterile and do not produce gametes, however, their gonads still contain autologous germ cells with which the transplanted cells need to compete. As an alternative, germ cell-deficient fish were produced by knocking down or knocking out the *dead end* gene^15–18^ which is crucial in maintaining the germ cell fate of primordial germ cells (PGCs)^17^. An additional challenge of the SSC transplantation strategy is the visualization of donor-derived germ cells within the recipient gonads. To this end, most studies utilized transgenic strains in which *vasa*-positive cells are fluorescently labeled^19,20^, therefore enabling the visualization of fluorescently-labelled donor cells within recipient gonads.

As SSC transplantation and surrogate production technology coupled with cryopreservation offer new possibilities in the conservation of valuable genetic resources, in the present study we have successfully developed methods for cryopreservation of zebrafish spermatogonia (through both slow-rate freezing and vitrification) and demonstrated the reproducibility of the presented protocols in several zebrafish lines. Additionally, we have produced functional sperm and viable progeny from cryopreserved spermatogonia trough germ cell transplantation.

## Results

### Optimization of the slow-rate freezing protocol

**Figure 1:**
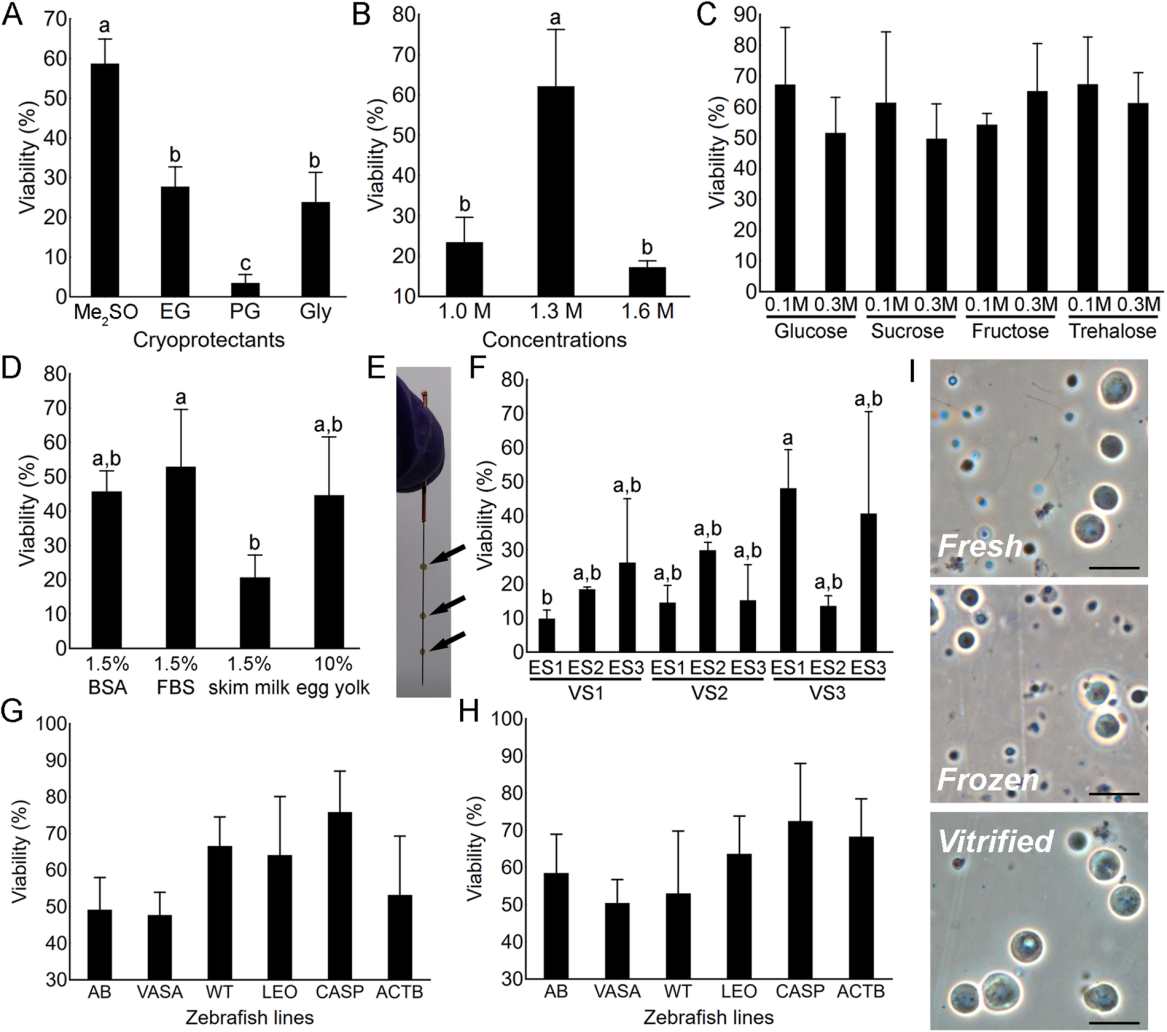
Optimization of the slow-rate freezing (**A**–**D**) and vitrification (**F**) protocols. (**A**) Viability of spermatogonia after freezing with 1.3 M dimethyl sulfoxide (Me_2_SO), ethylene glycol (EG), propylene glycol (PG) and glycerol (Gly) (N = 3). (**B**) Viability of spermatogonia after slow-rate freezing with either 1.0, 1.3 or 1.6 M of Me_2_SO (N = 3). The effects of sugar (**C**) and protein (**D**) supplementation of spermatogonia viability (N = 3). (**E**) Testes (arrows) pinned on an acupuncture needle for the needle-immersed vitrification (NIV) method. (**F**) The effects of different equilibration (ES) and vitrification (VS) solutions on spermatogonia viability after NIV (N = 3). Reproducibility of the developed freezing (**G**) and vitrification protocols (**H**) demonstrated on AB wild-type (AB), vasa (*ddx4*^*sa6158/sa6158*^; VASA), Wilms tumor (*wt1b*; WT), leopard (*gja5b*^*t1*^; LEO), casper (*mitfa*^*w2/w2*^*; mpv17*^*a9/a9*^; CASP) and β-actin (*pku341Tg*; ACTB) zebrafish lines. (**I**) Testicular cell suspensions prior to, and after cryopreservation. All values are presented as mean ± SD. Different letters above the SD bars indicate statistical significance (Tukey’s HSD, *p* < 0.05). Scale bars: (**I**) 20 µm.

The viability of spermatogonia frozen with the addition of 1.3 M dimethyl sulfoxide (Me_2_SO) in the cryomedium was significantly higher than the viability of those frozen with other tested cryoprotectants in the same concentration (Tukey’s HSD, *p* < 0.01; Fig. 1A). When testing the effects of different Me_2_SO concentrations, viability was significantly higher when cryopreserving with 1.3 M, compared to freezing with either 1.0 or 1.6 M (Tukey’s HSD, *p* < 0.01; Fig. 1B). The supplementation of cryomedium containing 1.3 M Me_2_SO with different sugars (glucose, sucrose, fructose and trehalose in 0.1 and 0.3 M) did not yield significant differences (Tukey’s HSD, *p* > 0.05; Fig. 1C), therefore we continued using 0.1 M trehalose in further trials. Finally, the addition of different protein fractions as non-permeating cryoprotectants (1.5% BSA, 1.5% FBS, 1.5% skim milk and 10% egg yolk) was assessed. Only the presence of 1.5% skim milk produced significantly lower germ cell viability (Tukey’s HSD, *p* < 0.01; Fig. 1D). Therefore, a cryomedium containing 35.2% extender, 1.3 M Me_2_SO, 0.1 M trehalose and 1.5% BSA was used in transplantation trials.

### Optimization of the vitrification protocol

Vitrification was conducted using needle immersed vitrification (NIV) by pinning three testes (as biological replications) on an acupuncture needle (Fig. 1E), immersing them into equilibration (ES) and vitrification solutions (VS), and subsequently plunging them directly into liquid nitrogen. The effectiveness of vitrification was assessed by testing three different ES and VS (yielding a total of nine ES/VS test groups) containing various amounts of methanol (MeOH), propylene glycol (PG) and Me_2_SO. Only the vitrification solutions had a significant effect on the testicular germ cell viability after warming (two-factor ANOVA, *p* < 0.01). The highest viability was obtained when combining VS3 containing lower concentrations of PG and Me_2_SO (3 M of both) with either ES1 (containing 1.5 M MeOH and 1.5 M PG; 48.04 ± 11.45%) or ES3 (containing 1.5 M PG and 1.5 M Me_2_SO; 40.69 ± 29.9%) (Fig. 1F). The combination of ES1 and VS3 was used in transplantation trials.

### Reproducibility of the cryopreservation protocols in different zebrafish transgenic lines

The reproducibility of the optimal freezing (containing 35.2% extender, 1.3 M Me_2_SO, 0.1 M trehalose and 1.5% BSA) and vitrification protocols (containing ES1: 1.5 M MeOH and 1.5 M PG and VS3: 3 M PG and 3 M Me_2_SO) was tested by cryopreserving whole testes of six different zebrafish lines (AB wild-type, casper (*mitfa*^*w2/w2*^*; mpv17*^*a9/a9*^), leopard (*gja5b*^*t1*^), *vasa::egfp* (*ddx4*^*sa6158/sa6158*^) transgenic line, *Wilms tumor::egfp* (*wt1b*) transgenic line and *β-actin:yfp* (*pku341Tg*) transgenic line). Both freezing and vitrification protocols proved to be reproducible since they yielded viability rates of nearly (or higher than) 50% (Fig. 1G,H). In the fresh samples and after freezing, early-stage germ cells were not the only cells present in the cell suspensions since numerous spermatids and spermatozoa were also present (Fig. 1I). However, after vitrification, the number of these cells significantly decreased and the cell suspensions were partly enriched for the early-stage germ cells.

### Transplantation and incorporation of the cryopreserved spermatogonia

**Figure 2:**
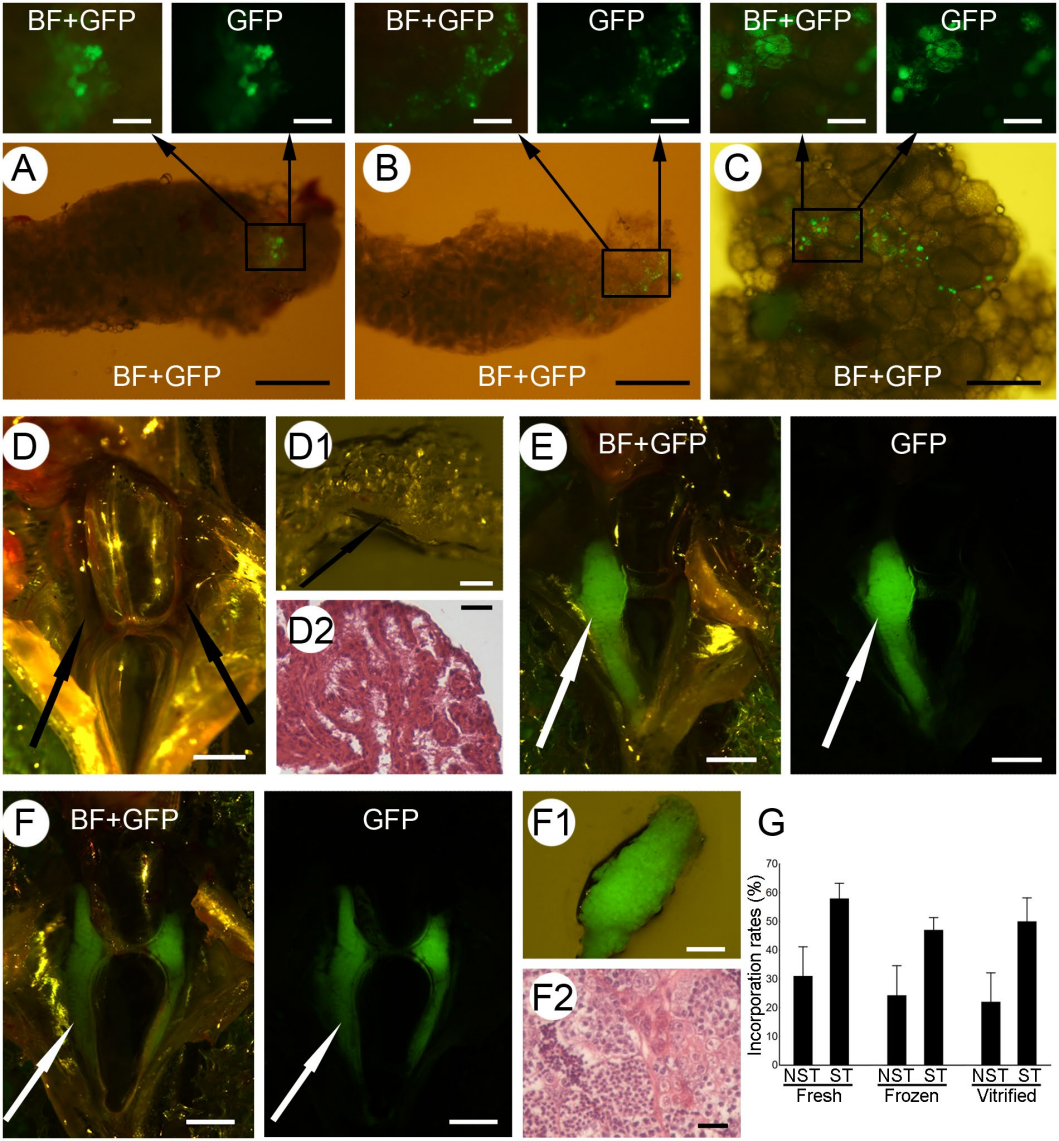
Incorporation and proliferation of fresh and cryopreserved spermatogonia. The incorporation and proliferation of fresh (**A**) and cryopreserved (**B**,**C**) *vasa::egfp* spermatogonia within the testes (**A**,**B**) and ovaries (**C**) of non-sterilized recipients. Testes (arrows) of the control MO-sterilized recipients (**D**) appear undeveloped under the stereomicroscope (**D1**), while the histological analysis (**D2**) displayed a lack of developing germ cells. Recipients of *actb:yfp* spermatogonia displayed either one (**E**) or both (**F**) developed testes (arrows). Developed testes displayed strong green fluorescence originating from donor *actb:yfp* spermatogonia (**F1**), while histological analyses displayed clear differentiation of germ cells into spermatozoa (**F2**). (**G**) Incorporation of fresh and cryopreserved spermatogonia into non-sterilized (NST) or MO-sterilized (ST) recipients. Values are presented as mean ± SD. Scale bars: (A, B, C) 500 µm; (top panels) 100 µm; (D, E, F) 1 mm; (D1, F1) 200 µm; (D2, F2) 20 µm.

To determine whether spermatogonia are functional and still have the capacity to colonize the recipient gonads and proliferate inside them after cryopreservation, fresh, frozen/thawed and vitrified/warmed spermatogonia from *vasa::egfp* transgenic line were transplanted into AB wild-type zebrafish larvae (7 dpf). Survival of the recipients was 85 ± 5%, similar to the untreated control larvae (80 ± 3%, *p* > 0.05). Recipients of fresh spermatogonia dissected 50 days after transplantation displayed a green fluorescent signal within their gonads indicating that donor cells had the ability to colonize the recipient gonads. Additionally, the large number of fluorescent cells forming colonies within the recipient gonads indicated that the donor cells were able to proliferate inside the recipient gonads (Fig. 2A–C). Similarly, frozen and vitrified spermatogonia also retained their migrating ability as they incorporated into the recipient gonads, but also retained their mitotic activity as they also proliferated within the recipient gonads, similarly to the fresh cells (Fig. 2B,C). Interestingly, transplanted spermatogonia incorporated and proliferated in gonads of both sexes, i.e. in both testes (Fig. 2A,B) and ovaries (Fig. 2C). The number of recipients containing incorporated donor-derived spermatogonia did not differ among the groups: 14 of 45 (31%) in the fresh control group; 11 of 45 (24%) in the frozen/thawed group; and 10 of 45 (22%) in the vitrified/warmed group (Fig. 2G).

After the initial transplantation assay, spermatogonia from *actb:yfp* transgenic line were transplanted into sterilized (by *dnd*-morpholino oligomer; MO1-*dnd*) wild-type AB larvae (7 dpf). Survival of injected embryos was comparable to the survival of the untreated controls (89% vs 80%). Upon reaching maturity, all recipient individuals morphologically appeared to be male. Dissection and subsequent histological analysis (six months after transplantation) of the MO-injected control individuals revealed no signs of germline development as the gonads were comprised of somatic cells only (Fig. 2D–D2). Dissection of recipient fish revealed that all developing gonads displayed green fluorescent signal corroborating the previously observed results that fresh, frozen/thawed and vitrified/warmed spermatogonia retain their ability to colonize and proliferate within recipient gonads (Fig. 2E–F2). Histological analysis of the gonads indicated that spermatogonia proliferated, differentiated and produced donor-derived spermatozoa (Fig. 2F2). As observed in the previous transplantation trial, the number of recipients containing incorporated donor-derived spermatogonia did not differ among the groups: 14 of 24 (58%) in the fresh control group; 9 of 19 (47%) in the frozen/thawed group; and 13 of 26 (50%) in the vitrified/warmed group (Fig. 2G). On average, 27% of recipients demonstrating successful incorporation and proliferation of donor cells had only one developed testis (Fig. 2E), while the rest displayed both testes developed (Fig. 2F). Expression of *yfp* was further confirmed by RT-PCR using RNA extracted from the resulting fluorescent testes.

### Production of gametes and donor-derived progeny using cryopreserved spermatogonia

**Figure 3:**
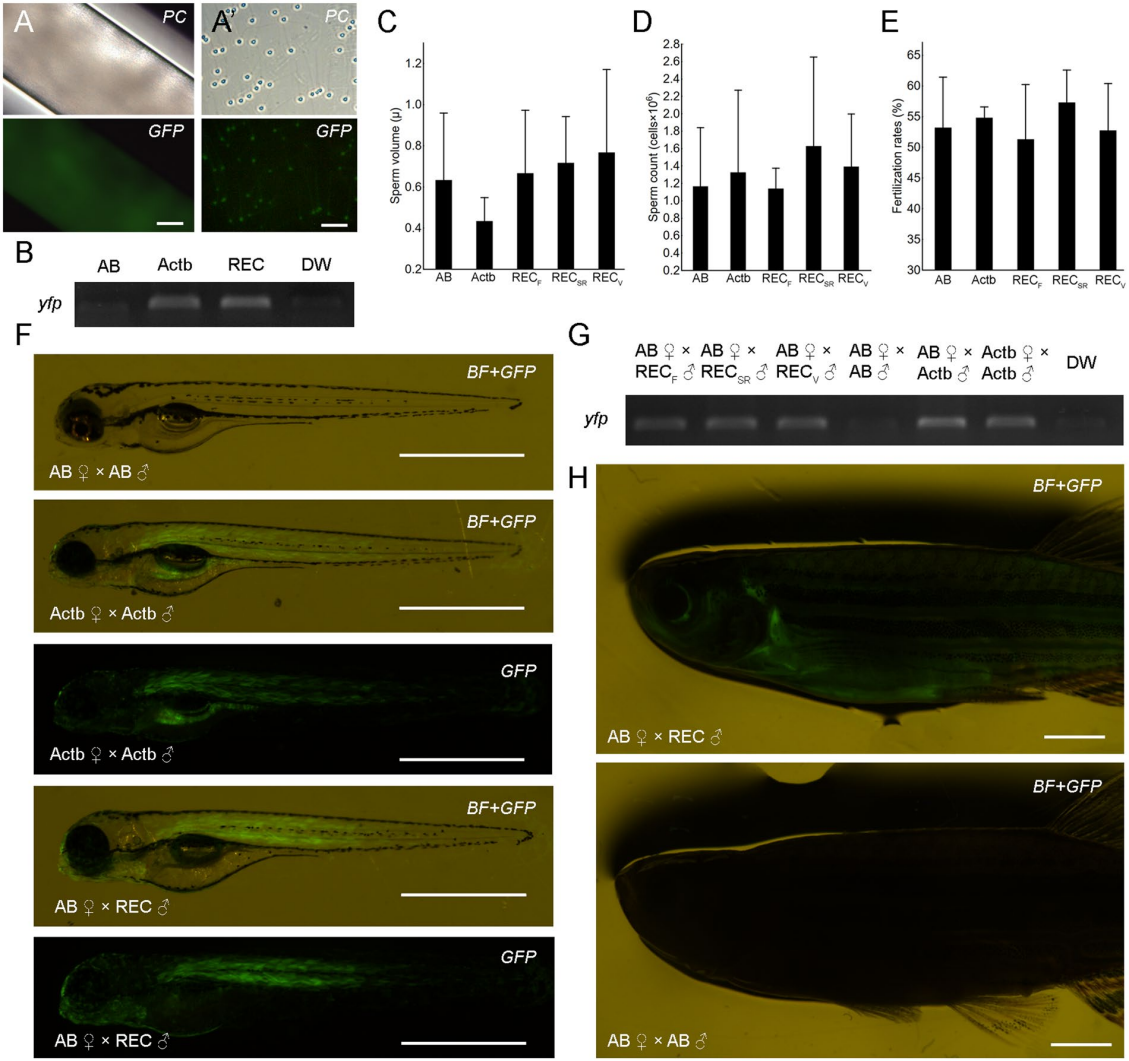
Production of donor-derived spermatozoa and progeny from MO-sterilized recipients. Milt (**A**) and individual spermatozoa (**A’**) stripped from recipients demonstrating yfp fluorescence. (**B**) RT-PCR amplification of *yfp* in milt obtained from wild-type AB (AB), β-actin transgenic (*pku341Tg*; Actb) and recipient (REC) zebrafish. Milt volume (**C**) and sperm count (**D**) of milt obtained from wild-type AB (AB), β-actin transgenic fish (*pku341Tg*; Actb) and recipients of fresh (REC_F_), frozen (REC_SR_) and vitrified (REC_V_) spermatogonia. (**E**) Fertilization rates after spawning control fish as well as recipient males and wild-type AB females. (**F**) Three dpf larvae obtained from crossing control fish and recipient males with wild-type AB females under fluorescent stereomicroscope. (**G**) PCR amplification of *yfp* in offspring obtained from control crossings, as well as from crossing recipient males with wild-type AB females. (**H**) F1 individuals developed normally and donor-derived individuals displayed fluorescent signal compared to the control crossing six months after fertilization. Values in **C**–**E** are presented as mean ± SD. Lack of different letters above SD lines indicate the lack of statistical significance (Tukey’s HSD, p > 0.05). Scale bars: (**A**) 100 µm; (**A’**) 20 µm; (**F**) 1 mm; (**H**) 2.5 mm.

Similarly to the colonization and proliferation rates, an average of 43% of sterilized recipients produced milt. Both obtained milt (Fig. 3A) and individual spermatozoa (Fig. 3A’) displayed a green fluorescent signal, which was additionally corroborated with positive RT-PCR amplification of *yfp* (Fig. 3B). Milt volume (Fig. 3C), sperm count (Fig. 3D) and kinematic properties of the spermatozoa (Supplement 1) did not significantly differ between the recipient fish and AB wilt type and *actb:yfp* control individuals. None of the sterilized control individuals produced any milt.

Upon reaching maturity (six months after transplantation), recipient fish were naturally mated with wild-type AB females to produce progeny. Fertilization rates were similar between all tested groups (Fig. 3E; Tukey’s HSD, *p* > 0.05). All produced larvae displayed green fluorescent signal similar to that of the *actb:yfp* larvae indicating that the larvae were of donor-derived origin (Fig. 3F). The expression of *yfp* in larvae obtained from mated recipient fish was additionally confirmed through RT-PCR amplification using total RNA extracted from the resulting larvae (Fig. 3G). Furthermore, all F1 individuals developed normally and displayed green fluorescence during the subsequent six months (Fig. 3H).

## Discussion

In the present study, we have successfully developed the cryopreservation methodology (through both freezing and vitrification) for the whole zebrafish testes by producing viable early-stage germ cells after thawing/warming. The recovered germ cells were physiologically functional since they were able to colonize the recipient gonads, proliferate within them and display production of functional gametes. Lastly, spawning of recipient males with wild-type females produced offspring carrying donor traits thus indicating their donor-derived origin. The protocols developed in this study can be applied to the reconstitution of whole zebrafish lines since testes of donor individuals can be crypreserved for theoretically indefinite periods, and subsequently transplanted into sterile individuals thus producing donor-derived gametes and offspring. Utilization of this technique would greatly alleviate the pressure for storage optimization created by current scientific practices and would offer a new perspective in long-term preservation of valuable zebrafish genetic resources.

Both freezing and vitrification protocols seem to be similarly effective in cryopreservation of zebrafish spermatogonia. Me_2_SO appeared to be superior to other cryoprotectants in freezing of SSCs similarly to the reports of Marinović *et al*.^21^, Linhartova *et al*.^22^, Lee *et al*.^12^. Even though higher cryoprotectant concentrations (2–3 M) are more favorable in cyprinid species such as tench^21^, goldfish^21^ and common carp^23^, zebrafish SSCs displayed higher viability at more moderate concentrations (of 1.3 M) similarly to the salmonid species^12,13^. This indicates that species-specific differences in optimal cryopreservation protocols exist, and that protocols need to be optimized for each species. Sugar and protein supplementation did not enhance the cryopreservation outcome most likely due to a significant amount of sugar and protein already circulating in the blood and extracellular compartments of the testes^24^. However, a dose-response study was not conducted for the protein supplementation, nor for particular extender components; therefore refinement of such parameters might further improve the viability rates observed in the present study.

The vitrification methodology used in this study has recently started to attract many cryobiologists as it outperforms freezing in several key aspects: (1) time-effectiveness since the preparation of samples is faster, several samples can be manipulated at the same time, and the cooling itself is radically faster; (2) low volumes of lN_2_ needed^25,26^. Needle immersed vitrification (NIV) is especially significant as it allows the manipulation of several tissue pieces at the same time, but also enables a direct contact between the tissue and liquid nitrogen thus increasing the cooling and warming rates as well as reducing the amount and concentration of cryoprotectants needed^26–28^. On the other hand, compared to the freezing protocols, vitrification does need significantly higher concentrations of cryoprotectants to enable the transition to the glassy amorphous state which in turn may be toxic to the cells^29^. Combining two cryoprotectants in similar concentrations enables the use of high overall cryoprotectant concentrations, however, each of them is in low enough amount to avoid toxicity to the cells^25^. The protocol using 3 M Me_2_SO and 3 M PG in the VS yielded the highest spermatogonia viability, similarly to the reports of Lujić *et al*.^25^. Additionally, vitrification displayed one significant advantage over the freezing protocols since far less spermatozoa were observed after vitrification. Most likely, spermatozoa do not survive the vitrification process, get digested during the enzymatic treatment and therefore the suspensions are enriched for spermatogonial stem cells.

Sterilization of the recipients generated an all-male population. Similar was reported by Slanchev *et al*.^15^, Li *et al*.^30^, Tzung *et al*.^16^, Gross-Thebing *et al*.^17^ where the knockdown or knockout of the *dead end* gene (that is involved in maintaining the germ-line fate of PGCs^17^) also produced all-male zebrafish populations since gonads adopt the testis fate in the absence of germ cells^31^. Germ line deficient testes have a normal somatic structure and are capable of supporting gametogenesis if exogenous germ cells are introduced as indicated by this and other studies^30,32^. Additionally, as such gonads have more available stem cell niches for the transplanted cells, the incorporation rates should be inherently higher in the germ line deficient recipients^32^. Contrary to the study of Li *et al*.^30^ which did not display such an event, in the present study transplantation into sterilized individuals yielded higher incorporation rates than the transplantation into non-sterilized wild-type individuals. Even though the exact reason for this difference between studies is ambiguous, the possible reason might lie in the methodology used for sterilization; the present study utilized morpholino oligonucleotides to inhibit translation of the *dnd* mRNA for a certain time window during which PGCs migrate, while in the study of Li *et al*.^30^ the knock out of *dnd* through ZFNs induced a lifetime absence of the gene. As observed by Škugor *et al*.^33^, *dnd*-KD results in several metabolic impairments, predominantly in the sex hormone metabolism. The lifetime absence of *dnd* may indeed have a profound influence on the proliferation and differentiation of incorporated donor cells, hence the very low (~5%) proliferation rate in the study of Li *et al*.^30^. Additionally, these metabolic impairments caused by the absence of *dnd* could be responsible for the unsuccessful hormonal induction into females in *dnd*-KO individuals after several trials, even though sex reversal in *dnd*-KD fish was reported as successful^15^.

Spawning of the recipient fish with wild-type females produced viable offspring demonstrating donor-derived traits. All F1 offspring displayed a green fluorescent signal under the microscope, expression of *yfp* was confirmed with RT-PCR amplification, and most importantly, F1 individuals developed and matured normally. Therefore, we can conclude that the offspring produced by mating of recipient males and wild-type females carried donor-derived traits. One important issue to factor in is that the offspring will be heterozygous for the assessed trait. Therefore, pure homozygous individuals can be obtained only in the F2 generation, where approximately 25% of the F2 offspring will be homozygous for the given trait. This can be circumvented by transplanting spermatogonia into both sterilized and non-sterilized recipients. Spermatogonia have been demonstrated to develop into functional eggs when transplanted into female recipients^12,14^, which was also demonstrated in the present study. Therefore, by spawning sterilized recipient males with non-sterilized recipient females, a certain percentage of F1 offspring will be homozygous for the assessed trait. Such fish can then be selected and used for producing the F2 generation which will be 100% homozygous for the given trait. Lastly, hormonal induction of *dnd*-KD sterilized individuals into females needs to be confirmed and tested. Preliminary results obtained by Saito *et al*. (cited from Goto and Saito^34^) display that eggs can be obtained from sterilized zebrafish recipients after hormonal treatment. In such case, the production of 100% donor-derived eggs, and subsequently 100% donor-derived and homozygous offspring would be possible.

The strategy for the preservation of genetic resources developed in this study is complementary to the sperm cryopreservation techniques currently utilized in leading zebrafish line repositories as well as other zebrafish facilities^10^. A key difference between the two is that freezing of testes requires sacrificing donor individuals, while sperm cryopreservation enables a long-term utilization of one individual male. Therefore while some individuals can be kept for milt production, several males (or in need even the whole line) can be sacrificed so that the genetic resource can be recovered at a later date. If we take into account that approximately 50% of the SSCs survive the cryopreservation procedure, and that donor SSCs incorporate into 30–50% of the recipient individuals, approximately 10–15 recipients producing donor-derived gametes can be obtained from sacrificing one male. Additionally, even though the application of sperm cryopreservation has been proven in practice^35^, the application of this method only preserves the male germline, therefore, the line needs to be recovered usually from the AB wild-type eggs. In this case, the F1 generation is 100% heterozygous, while in the F2 generation only 25% of the fish are homozygous, thus making recovery of the homozygous lines laborious and expensive. Conversely, the surrogate preservation technique allows for the full homozygous line to be recovered in the F1 generation spawned from the recipients. As described above, if the hormone-induced feminization of *dnd*-KD proves to be successful, 100% of the F1 individuals will be donor-derived and homozygous thus greatly decreasing the labor as well as time needed to produce homozygous individuals from the cryopreserved sperm. As an alternative, cryopreservation of oocytes could be used for conservation of female genetic resources, however, only immature oocytes (especially stage I and II oocytes) display favorable survival and their physiologic activity is very limited as they display lower growth and maturation potential after cryopreservation compared to fresh controls^36,37^.

## Material and Methods

All experiments performed in Japan were conducted in in accordance to the Guide for the Care and Use of Laboratory Animals, Tokyo University of Marine Science and Technology, while all experiments performed in Hungary were conducted in accordance with the Hungarian Animal Welfare Law, Hungarian Government Directive 40/2013 on Animal Experimentation and the Directive 2010/63/EU of the European Parliament and of the Council. In addition, all experiments conducted in Hungary were approved under the Hungarian Animal Welfare Law (approval number: PE/EA/188-6/2016).

### Animal husbandry and tissue preparation

Animals kept at the Tokyo University of Marine Science and Technology used for the optimization of the freezing protocol and for testing the functionality of the cryopreserved cells were reared under a 14-h/10-h light/dark photoperiod at 28 °C. Fish were fed 2–3 times a day with a commercial diet (Hikari Labo 130 [Kyorin, Hyougo, Japan]; and daily with Otohime A, B1, and B2, [Nisshin Marubeni Feed, Tokyo, Japan]) and *Artemia* nauplii.

Animals kept at the Szent István University used for the optimization of the vitrification protocol, testing the reproducibility of the protocols and obtaining offspring from transplanted cells were reared in a recirculating system (Tecniplast Zebtec, Tecniplast, Buguggiate, Italy). Fish were kept under a 14-h/10-h light/dark photoperiod at 25 °C and were fed twice a day with SDS Small Gran granulated feed and daily with *Artemia* nauplii.

Three to six month old males were euthanized with a 2-phenoxyethanol overdose. Testes were aseptically removed and placed in Leibovitz L-15 medium supplemented with 10% FBS on ice until further analyses (max 30 min).

### Slow-rate freezing of the whole testes

Optimization of the slow-rate freezing protocol was conducted in four sequential experiments similarly to Lee *et al*.^12^ for rainbow trout where in each experiment one cryopreservation parameter was changed and the best outcome was used in the subsequent experiment. Firstly, four cryoprotectants (dimethyl sulfoxide – Me_2_SO, propylene glycol – PG, ethylene glycol – EG and glycerol – Gly) in 1.3 M were tested. Afterwards, three Me_2_SO concentrations of 1.0 M, 1.3 M and 1.6 M were tested. Lastly, sugar (glucose, trehalose, sucrose and fructose in 0.1 and 0.3 M) and protein (1.5% BSA, 1.5% FBS, 1.5% skim milk and 10% egg yolk) supplementation was tested. Whole testes of the *vasa::egfp* transgenic zebrafish (*ddx4*^*sa6158/sa6158*^) were transferred into 1.8 ml cryotubes containing 500 µl of cryomedium comprised of 35.2% extender (100% extender: 55.27 mM HEPES, 375.48 mM NaCl, 7.28 mM KCl, 23.10 mM KH_2_PO_4_, 3.82 mM Na_2_HPO_4_, 3.64 mM sodium pyruvate, 2.6 mM CaCl_2_·2H_2_O and 1.4 mM MgCl_2_·6H_2_O, pH 7.8^12^) and appropriate amount of cryoprotectants, sugar and protein supplementation. Testes were equilibrated in the cryomedium for 20 min on ice. Cryotubes were then placed into BiCell (Nihon Freezer) or CoolCell (BioCision) freezing containers and placed into a deep freezer enabling the cooling rates of ~1 °C/min for 1.5 h (−80 °C) before being plunged into the liquid nitrogen. After at least 1 day of storage, cryotubes were thawed in a 25 °C water bath for at least 2 min and the testes were rehydrated in three changes of L-15 supplemented with 10% FBS.

### Vitrification of the testes

Optimization of the vitrification protocol was conducted by testing the effects of three different equilibration solutions and three different vitrification solutions on spermatogonia viability similarly to Lujić *et al*.^25^ for brown trout oogonia. Equilibration (ES1 – ES3) and vitrification (VS1 – VS3) solutions contained different combinations and concentrations of Me_2_SO, MeOH and PG (ES1: 1.5 M MeOH + 1.5 M PG; ES2: 1.5 M MeOH + 1.5 M Me_2_SO ES3: 1.5 M PG + 1.5 M Me_2_SO; VS1: 1.5 M MeOH + 4.5 M PG; VS2: 1.5 M MeOH + 5.5 M Me_2_SO; VS3: 3 M PG + 3 M Me_2_SO) while the extender used consisted of L-15 supplemented with 10% FBS, 25 mM HEPES and 0.5 M trehalose. Vitrification of testes was conducted by needle-immersed vitrification (NIV) following the protocol described by Marinović *et al*.^26^. Namely, testes of wild-type AB zebrafish were pinned to an acupuncture needle and incubated in each equilibration solution for 5 min and in each vitrification solution for 30 s. Excess liquid was carefully absorbed from the tissue by a sterile paper towel and the needles were plunged in liquid nitrogen. After at least one day of storage, tissues were warmed in three sequential warming solutions containing L-15 supplemented with 10% FBS and various concentrations of sucrose (WS1 – 3 M; WS2 – 1 M; WS3 did not contain sucrose).

### Assessment of spermatogonia viability

During the cryopreservation protocol optimization, one (left) testis of each fish was used as a fresh control and was immediately dissociated, while the other (right) testis was cryopreserved and subsequently dissociated after thawing/warming. Testes were dissociated in a solution of L-15 medium containing 2 mg/ml collagenase, 1.5 mg-ml trypsin and 30 µg/ml DNase for 1.5 h on a shaking plate at 28 °C. The dissociation process was stopped by adding 10% FBS (v/v), filtering through 30 µm filters and centrifuging at 200 × g. Viability of cells within the suspension was verified by the trypan blue exclusion test. The exception was when using the *vasa:egfp* line since only live spermatogonia emit fluorescence, while dead spermatogonia are dissociated and lose their fluorescence. As the total number of cells obtained from the two testes did not significantly differ (1 ± 0.5 × 10^5^ vs 1.1 ± 0.7 × 10^5^; one-way ANOVA, *p* > 0.05), viability was assessed as the proportion of the total number of cells obtained from the cryopreserved testis compared to the total number of cells obtained from the fresh testis: ${Viability}( \% )=({N}_{cryopreserved}/{N}_{fresh})\times 100.$

### Reproducibility of the developed protocols

The reproducibility of the optimized freezing and vitrification protocols was tested by cryopreserving testicular tissue of zebrafish from six different lines: (1) AB wild-type, (2) casper (*mitfa*^*w2/w2*^*; mpv17*^*a9/a9*^), (3) leopard (*gja5b*^*t1*^), (4) *vasa::egfp* (*ddx4*^*sa6158/sa6158*^) transgenic line, (5) *Wilms tumor::egfp* (*wt1b*) transgenic line and (6) *β-actin:yfp* (*pku341Tg*) transgenic line. All transgenic lines have been bred on the AB wild-type line for at least five generations in our laboratory. Testes were dissected and cryopreserved as described above. The optimal freezing medium contained 35.2% extender, 1.3 M Me_2_SO, 0.1 M trehalose and 1.5% BSA, while the optimized vitrification media included ES1 (1.5 M MeOH and 1.5 M PG) and VS3 (3 M PG and 3 M Me_2_SO). As previously described, one testis of each fish was used as a fresh control, while the other was frozen; viability was assessed as above stated. All experiments were conducted in triplicates.

### Transplantation of spermatogonia

Testes (fresh, frozen/thawed and vitrified/warmed) of donor zebrafish (either *vasa::egfp* or *actb:yfp*) were dissociated to isolate early-stage germ cells. Approximately 200 nl containing 3000 fluorescent early-stage germ cells were transplanted into the abdominal cavity of 7-dpf larvae^30^. Recipient larvae were placed in fresh system water and reared at 28 °C. Firstly, the transplantation study was conducted on non-sterilized wild-type AB zebrafish larvae. Recipients were reared until 50 days post-transplantation, dissected and the gonads were checked for fluorescent signal under a BX-53 epifluorescent microscope (Olympus).

The second transplantation study was conducted by using wild-type AB zebrafish larvae sterilized by utilizing morpholino oligonucleotide injection against the *dead end* gene (MO1-*dnd*; 5′-GCTGGGCATCCATGTCTCCGACCAT-3′^38^). Approximately 3 ng of MO1-*dnd* dissolved in nuclease free water was injected into 1- to 4-cell stage embryos. Seven days after fertilization, fluorescently labelled early-stage germ cells were transplanted into the sterilized embryos as previously described. Recipient fish were reared until sexual maturity when they were dissected (6 months after transplantation) and the gonads were checked for fluorescent signal under a Leica M205FA stereomicroscope. Developing gonads displaying fluorescent signal, as well as undeveloped gonads were collected for histology by fixing in Bouin’s solution. Samples were routinely processed, embedded in paraffin, sectioned at 3 µm thickness and stained by standard H&E staining method. Additionally, gonads were sampled in TRI reagent (Molecular Research Center) for *yfp* expression analysis.

### Progeny tests

Since all recipient fish were males upon reaching maturity, recipients were stripped by abdominal massage and sperm was collected in glass capillaries to check for volume, spermatozoa number, as well as kinematic properties of the donor-derived spermatozoa. All spermatozoa were diluted 10 × in Hanks’ balanced salt solution (HBSS); sperm numbers were counted under the Burker-Turk type chamber; kinematic properties of stripped spermatozoa were assessed by a Computer Assisted Sperm Analysis software (CASA; SpermVision version 3.7.4, Minitube, Tiefenbach, Germany).

Offspring were produced by naturally mating mature recipient fish with wild-type females. Fertilization rates were calculated 24 hours post fertilization (hpf), while hatching rates were counted 48 hpf. Produced offspring were checked for fluorescent signal under a Leica M205FA fluorescent stereomicroscope 3 days post fertilization (dpf) and six months post-fertilization. Additionally, approximately 20 larvae per cryopreservation group were sampled in TRI reagent and the expression of *yfp* was verified.

### *yfp* expression analyses

To confirm the incorporation and proliferation of donor spermatogonia and the donor-derived origin of the produced offspring, testes, milt and larvae were sampled in TRI reagent as above stated. RNA was isolated according to the standard manufacturer’s protocol. RNA was reverse transcribed using the RevertAid first strand cDNA synthesis kit (ThermoFisher Scientific). The PCR amplification of *yfp* cDNA was conducted by using HOT FIREPol EvaGreen qPCR supermix (Solis BioDyne) and the following primers: YFP-forward: 5′-CTCGTGACCACCTTCGGCT-3′; YFP-reverse: 5′-TCCTGGACGTAGCCTTCGG-3′^39^. Thermal conditions were the following: 95 °C for 12 min for the initial denaturation and 30 cycles using 95 °C for 30 sec, 60 °C for 30 sec and 72 °C for 20 sec. Expected amplicon size was 107 bp.

### Statistical analysis

All values are presented as mean ± standard deviation (SD). All percentage data were log ratio-transformed prior to statistical analysis. One-way ANOVA followed by Tukey’s HSD post-hoc test was used to test the effects of different cryopreservation parameters on post-thaw viability of spermatogonia. Two-factor ANOVA followed by Tukey’s HSD post-hoc test was used to assess the effects of different equilibration and vitrification solutions on spermatogonia viability. All statistical analyses were conducted in Statistica 13.1 software (TIBCO software Inc., Palo Alto, CA, USA).

## Supplementary information

Supplement 1
