# Supplementary material for: Preservation of zebrafish genetic resources through testis cryopreservation and spermatogonia transplantation
Source: Sci Rep. 2019 Sep 25;9:13861. doi: 10.1038/s41598-019-50169-1 (PMC6761286; doi:10.1038/s41598-019-50169-1)

Preservation of zebrafish genetic resources through testis cryopreservation and spermatogonia transplantation

Zoran Marinović[1†], Qian Li[2†], Jelena Lujić[1*], Yoshiko Iwasaki[2], Zsolt Csenki[1], Béla Urbányi[1], Goro Yoshizaki[2‡], Ákos Horváth[1‡]

[1]Department of Aquaculture, Szent István University, Páter Károly u. 1., H-2100 Gödöllő, Hungary
[2]Department of Marine Biosciences, Tokyo University of Marine Science and Technology, 108-8477 Tokyo, Japan

[†]Authors contributed equally to the manuscript
[‡]Authors contributed equally to the manuscript
*Corresponding author

Supplement 1. Kinematic properties of spermatozoa obtained from wild type AB (AB), β-actin [Tg(*actb::eGFP*)] (Actb) zebrafish as well as from recipients of fresh (REC_F), slow-rate frozen (REC_{SR}) and vitrified (REC_V) spermatogonia.

	tMOT (%)	pMOT (%)	VCL (μm/s)	VAP (μm/s)	VSL (μm/s)	STR (%)	LIN (%)	WOB (%)	ALH (μm)	BCF (Hz)
AB control	79.2±16.4	57.3±22.2	76.0±12.9	66.9±12.4	56.9±10.2	0.84±0.03	0.74±0.04	0.87±0.02	1.4±0.2	27.9±1.9
Actb control	80.4±13.5	60.1±13.5	76.1±12.8	67.8±13.7	57.5±13.0	0.84±0.04	0.74±0.07	0.88±0.04	1.4±0.3	27.2±2.2
REC_F	77.4±6.9	56.7±7.5	73.0±7.7	66.4±8.2	55.2±8.2	0.82±0.04	0.75±0.05	0.90±0.02	1.2±0.2	29.6±3.1
REC_{SR}	72.6±20.7	48.1±15.0	69.0±8.9	60.2±8.8	48.5±9.0	0.79±0.04	0.69±0.05	0.86±0.02	1.4±0.2	28.9±2.5
REC_V	84.5±9.1	58.3±16.3	73.5±8.9	64.4±7.6	54.2±7.5	0.84±0.04	0.73±0.08	0.87±0.02	1.5±0.2	27.7±2.9

tMOT – total motility; pMOT – progressive motility; VCL – curvilinear velocity; VAP – average path velocity; VSL – straight-line velocity; STR – straightness; LIN – linearity; WOB – wobble; ALH – lateral head displacement; BCF – frequency of head displacement.

Supplementary information: Full-length gel

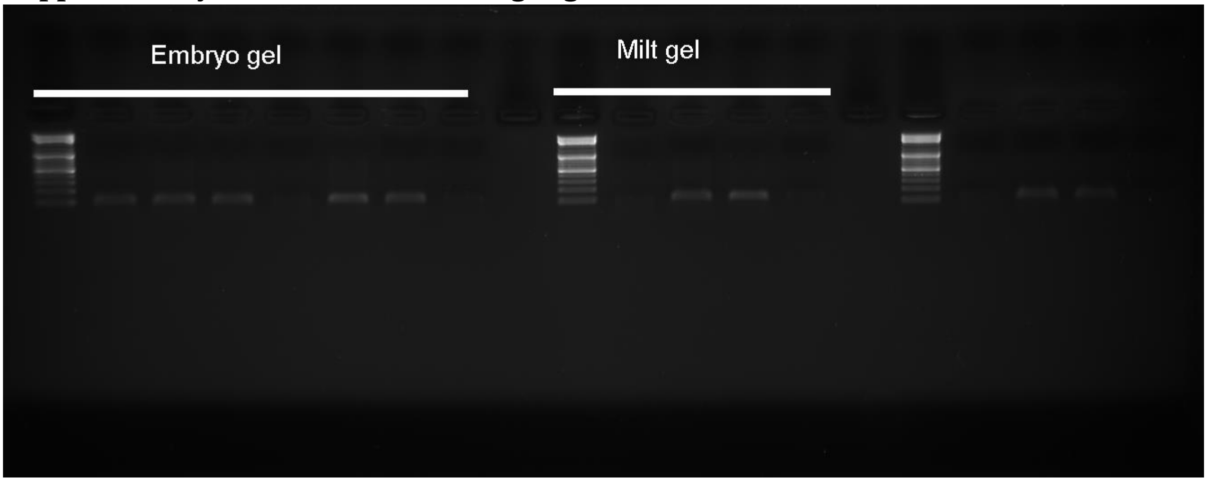

Supplement: Supplementary file 1 — Supplement 1 [file 41598_2019_50169_MOESM1_ESM.pdf]
